# Comparative Peptidomics Analysis of Fermented Milk by *Lactobacillus delbrueckii* ssp. *bulgaricus* and *Lactobacillus delbrueckii* ssp. *lactis*

**DOI:** 10.3390/foods10123028

**Published:** 2021-12-06

**Authors:** Hongji Ye, Xinyi Zhang, Yang Jiang, Min Guo, Xiaoming Liu, Jianxin Zhao, Hao Zhang, Wei Chen

**Affiliations:** 1 State Key Laboratory of Food Science and Technology, Jiangnan University, Wuxi 214122, China; 6190112165@stu.jiangnan.edu.cn (H.Y.); zhaojianxin@jiangnan.edu.cn (J.Z.); zhanghao61@jiangnan.edu.cn (H.Z.); chenwei66@jiangnan.edu.cn (W.C.); 2 School of Food Science and Technology, Jiangnan University, Wuxi 214122, China; lalazhang4516@163.com (X.Z.); 7160112009@vip.jiangnan.edu.cn (Y.J.); guomin@jiangnan.edu.cn (M.G.); 3 International Joint Research Laboratory for Pharmabiotics & Antibiotic Resistance, Jiangnan University, Wuxi 214122, China; 4 National Engineering Research Centre for Functional Food, Wuxi 214122, China; 5 Wuxi Translational Medicine Research Center, Jiangsu Translational Medicine Research Institute Wuxi Branch, Wuxi 214122, China

**Keywords:** *Lactobacillus delbrueckii*, *bulgaricus*, *lactis*, comparative peptidomics, digestion, bioactive peptide

## Abstract

Few studies have investigated the peptidomics of fermented milk by *Lactobacillus delbrueckii*. The aim of the present study was to interpret the peptidomic pattern of the fermented milk by five strains of *L. delbrueckii* ssp. *bulgaricus* and ssp. *lactis* prior to and after the simulated gastrointestinal digestion in vitro. The results indicated variations in the peptidomics among the samples, particularly between the samples of different subspecies. The peptides originating from β-casein were abundant in the samples of ssp. *bulgaricus*, whereas the peptides derived from αs1-casein and αs2-casein were more likely to dominate in those of ssp. *lactis*. For β-casein, the strains of ssp. *bulgaricus* displayed extensive hydrolysis in the regions of (73–97), (100–120), and (130–209), whereas ssp. *lactis* mainly focused on (160–209). The digestion appears to reduce the variations of the peptidomics profile in general. Among the five strains, *L. delbrueckii* ssp. *bulgaricus* DQHXNS8L6 was the most efficient in the generation of bioactive peptides prior to and after digestion. This research provided an approach for evaluating the peptide profile of the strains during fermentation and digestion.

## 1. Introduction

*Lactobacillus delbrueckii*, one of the thermophilic lactic acid bacteria (LABs), plays an important role in food fermentation, including ssp. *bulgaricus* and ssp. *lactis*, two subspecies isolated from milk niches. Previous studies showed that there were differences in carbohydrate metabolism between ssp. *bulgaricus* and ssp. *lactis* [1,2]. However, the characteristics of the peptidomic pattern and the profile of the bioactive peptides of the fermented milk from these two subspecies, as well as the influence of gastrointestinal digestion, have not been thoroughly investigated.

The proteolytic system provides peptides and free amino acids for the growth of strains during fermentation [3], and the cell-envelope proteinase (CEP) of LAB is essential for the hydrolysis of milk proteins [3,4,5]. The CEP of *L. delbrueckii* ssp. *bulgaricus* and *lactis* were classified as PrtB [6] and PrtL, respectively, with a unique amino acid substitution for substrate specificity at position 222 for PrtL and different hydrolysis patterns of the chromophoric peptides compared to PrtB [3]. How PrtB and PrtL from the two subspecies affect the peptide profile and generation of bioactive peptides of fermented milk has not been reported yet.

Milk fermentation by LAB is known for the generation of bioactive peptides [7,8] with different biological functions such as angiotensin-converting enzyme (ACE) inhibitors, antihypertensives, anti-inflammatories, antioxidants, and immunomodulators [9]. In particular, the lactotripeptides VPP and IPP were identified as ACE inhibitors and displayed the ability to reduce blood pressure in vivo; VPP and IPP have been detected in fermented milk by *L. helveticus* and could resist gastrointestinal digestion [10,11,12,13]. However, few studies have been conducted on the profile of peptides in fermented milk *L. delbrueckii* ssp. *bulgaricus* and *lactis*. Moreover, as gastrointestinal digestion affects the absorption and function of peptides, investigating the changes of the peptide profile during digestion are critical for evaluating the function of fermented milk.

Therefore, in the current study, the peptidomics of fermented milk by five strains of *L. delbrueckii* ssp. *bulgaricus* and ssp. *lactis* were investigated before and after the simulated gastrointestinal digestion in vitro in order to understand the pattern of peptide formation during fermentation and digestion.

## 2. Materials and Methods

### 2.1. Microorganisms and Culture Conditions

Three *L. delbrueckii* ssp. *bulgaricus* strains, DXJLHTS2M2, DQHXNS8L6, and 2038 and two *L. delbrueckii* ssp. *lactis* strains, D11M188 and ATCC12315, were used to ferment skim milk. All the strains were obtained from the Culture Collections of Food Microbiology, Jiangnan University (Wuxi, China).

### 2.2. Fermentation of Skim Milk

Fermented skim milks were manufactured using the previously referred to method with some modifications [14]. Skim milk was prepared by reconstituting 11% (*w/w*) skim milk powder. The bacterial strains were incubated into skim milk with an initial culture concentration of 1–5 × 10^6^ CFU/mL and incubated at 37 °C for 12 h. The viable cell counts of *L. delbrueckii* strains were determined using the plating method. Then, 0.5 mL samples were added to 4.5 mL of 0.9% (*w/v*) sterile saline and diluted serially as appropriate. Plates were incubated at 37 °C for 48 h under anaerobic conditions. Colonies were counted on each plate, and live-cell counts were expressed as lg(cfu∙mL^−1^). The pH was measured by a pH meter (Model 3100, Ohaus, Parsippany, NJ, USA). The experiments were conducted in three replications.

### 2.3. Preparation of Whey Fraction

Whey fraction of samples was prepared according to the previous method with moderate modifications [15]. The pH of 35 mL of the samples was adjusted to 4.6 and the supernatants were harvested after centrifugation at 10,000× *g* for 10 min at 4 °C and filtration with 0.45 µm nylon syringe filter. Then, ultrafiltration was applied using a membrane with a cut-off value of 10 kDa (EMD Millipore, Billerica, MA, USA), and the whey was stored at −20 °C prior to further analysis.

### 2.4. Simulated Gastrointestinal Digestion

The digestion process was conducted according to the previous study with slight modification [16]. The pH values of whey samples were adjusted to 2.0, and pepsin (Sigma Aldrich, St. Louis, MA, USA) was added at a ratio of 1:50 (*w/w*) enzyme to substrate. After digestion at 37 °C for 2 h, the pH was adjusted to 8 with 0.4 M NaOH, and then trypsin (Sigma Aldrich, St. Louis, MA, USA) was added to digest at 37 °C for 2 h. Then, samples were heated at 95 °C for 10 min to inactivate the enzyme activity. The supernatants of the digested samples were collected by centrifuge at 14,000× *g* for 5 min at 4 °C and filtered by a 0.45 µm nylon syringe filter. The digested samples were finally stored at −20 °C prior to further analysis.

### 2.5. Determination of Peptide Content

The peptide content of the samples was determined using Pierce^TM^ Quantitative Colorimetric Peptide Assay (Thermo Fisher Scientific, Waltham, MA, USA). The absorbance was read at 480 nm and the results were expressed as mg∙mL^−1^.

### 2.6. Identification of Peptides Sequence by UPLC-ESI-MS/MS

One milliliter of the samples was purified using C18 solid-phase extraction with Hypersep C18 100 mg microcolumns (Thermo Fisher Scientific, Waltham, MA, USA) and dried by centrifugal evaporation (miVac Centrifugal Vacuum Concentrators, Gene Vac, Ipswich, UK) for 2 h at 40 °C. Dried samples were dissolved in mobile phase A (MilliQ-treated water with 0.1% FA and 2% acetonitrile) and diluted to 0.5 mg∙mL^−1^. After being centrifuged for 10 min at 8000× *g*, supernatants were collected, and 2 µL were injected to an EASY nLC 1200 (Thermo Fisher Scientific, Waltham, MA, USA) with Acclaim PepMap^TM^ RSLC (50 μm × 15 cm, 2 µm, 100 Å, Thermo Fisher Scientific, Waltham, MA, USA). The linear gradient of mobile phase B (acetonitrile with 0.1% FA and 10% MilliQ-treated water) with flow rate of 0.2 µL∙min^−1^ was performed as follows: 6–20% for 40 min, 20–32% for 12 min, 32–80% for 4 min.

The chromatography device was coupled to a Q-Exactive mass spectrometer (Thermo Fisher Scientific, Waltham, MA, USA), and the liquid chromatography (LC) eluent was electrosprayed from the end of the column at an applied voltage of 2.3 kV. MS analysis was performed in sensitivity, positive ions, and data-dependent analysis (DDA) modes, and MS data were collected in the 150–2000 *m/z* range with a scan time of 0.2 s. A maximum of 15 precursor ions with an intensity threshold of 10,000 counts was selected for ion trap collision-induced dissociation (CID) fragmentation and subjected to collision energy ramping from 8 V to 9 V for low mass and 40 V to 90 V for high mass. The MS/MS spectra were recorded on the 180 to 2000 *m/z* range with a scan time of 0.1 s.

Database searches via Maxquant (1.5.2.8; Available online: https://maxquant.net/maxquant/ (accessed on 5 December 2014)) were performed using the UniProt databases (Available online: https://www.uniprot.org/ (accessed on 5 January 2021)) restricted to *Bos taurus* organism. A mass tolerance of 35 ppm and 3 missing cleavage sites as well as an MS/MS tolerance of 0.02 Da were allowed. Variable methionine oxidation was also considered. The relevance of protein and peptide identities was judged according to their identification generated by PEAKS Studio 7.0 (*p* < 0.05) and a false discovery rate < 1%.

### 2.7. Statistical Analysis

The mean ± standard deviation (SD) was used for the presentation of data, and multiple ANOVA was carried out with *p* < 0.05 considered a significant difference. Log10 scale transformation was used to carry out the statistical analysis of peptidomic, and principal component analysis (PCA) and partial least squares discriminant analysis (PLS-DA) were used, respectively, to visualize differences in peptidomic profiles between all samples and two subspecies groups in MetaboAnalyst (available online: https://www.metaboanalyst.ca (accessed on 30 September 2021)). Peptides detected in at least two of the three replicates were considered identified in samples, and the variable importance in projection (VIP) score was used to represent differences between two subspecies. The bioactivity of detected peptides was matched to the Milk Bioactive Peptide Database (available online: http://mbpdb.nws (accessed on 9 August 2021)). GraphPad Prism 8.0 (GraphPad Software, San Diego, CA, USA) was chosen to draw the figure.

## 3. Result and Discussion

### 3.1. Peptide Profile Analysis Revealed Variation among the Samples

**Figure 1:**
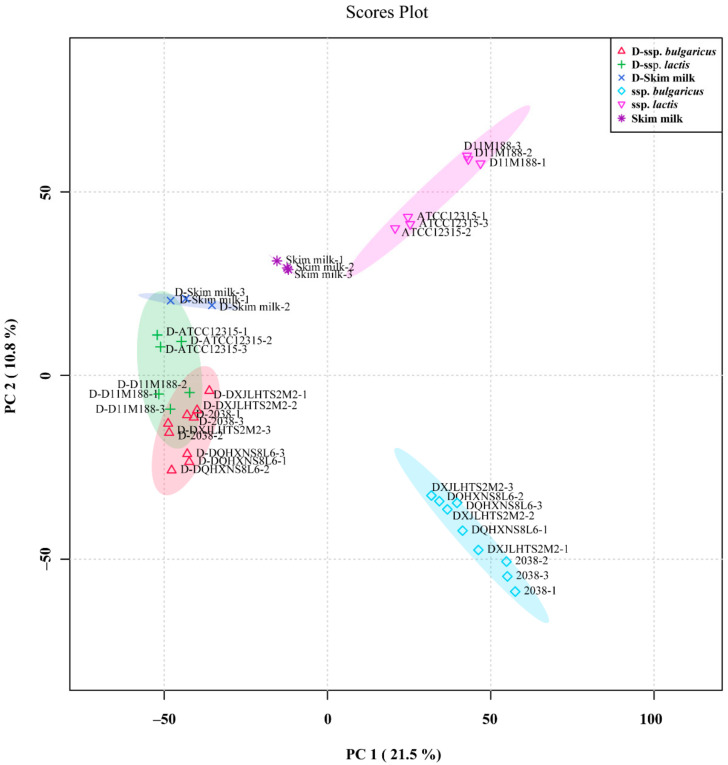
Principal component analysis for peptidomics of skim milk and fermented milks by *Lactobacillus delbrueckii* ssp. *bulgaricus* and *L. delbrueckii* ssp. *lactis* after 12 h of fermentation at 37 °C before and after the simulated gastrointestinal digestion in vitro.

The peptidomic structural differences of the skim milk, fermented milk samples, and digested samples are shown in Figure 1. For the samples of fermented milk, discrimination in the samples of two subspecies was observed, with skim milk clustered close to that of *L. delbrueckii* ssp. *lactis*, whereas the digestion appears to reduce the extent of discrimination of the fermented samples. The peptide content of the fermented milk of ssp. *bulgaricus* was generally higher than that of ssp. *lactis,* with the highest peptide content detected in the fermented milk of *L. delbrueckii* ssp. *bulgaricus* DXJLHTS2M2 and 2038 followed by DQHXNS8L6. (Figure S1), and variation in the pH value and the viable count was recorded among the samples (Table S1).

### 3.2. Comparative Peptidomic Analysis of Fermented Milk

**Figure 2:**
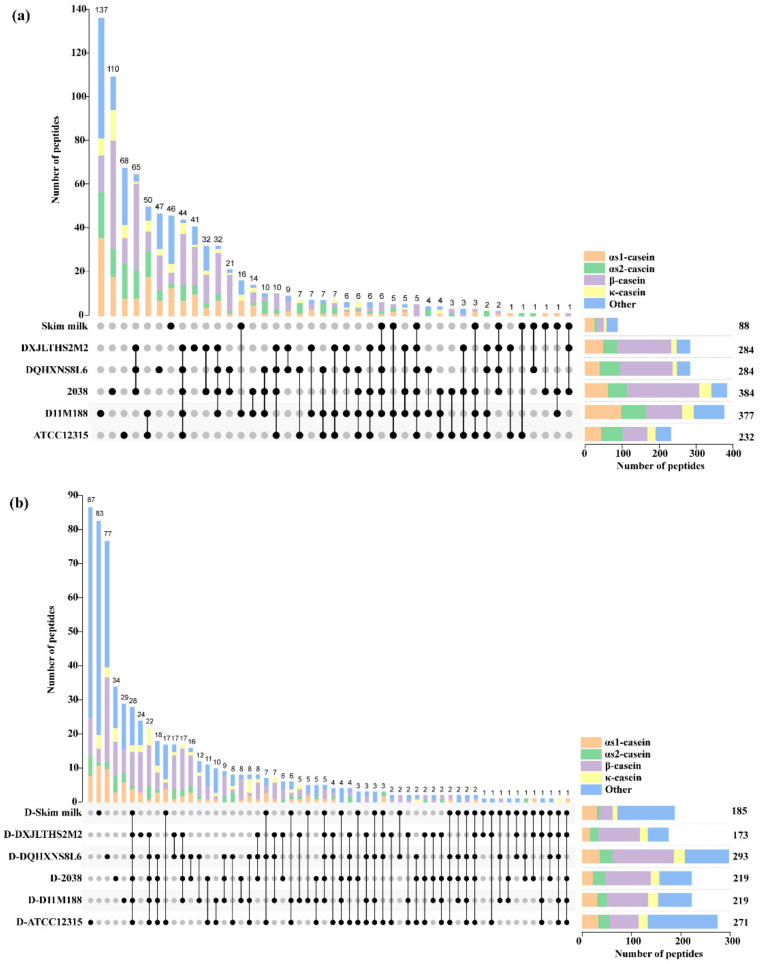
Upset plot showing the common and differential peptides among the fermented milk samples by *Lactobacillus delbrueckii* ssp. *bulgaricus* and *L. delbrueckii* ssp. *lactis* after 12 h of fermentation at 37 °C. (**a**) Common and differential peptides of the samples before digestion. (**b**) Common and differential peptides of the samples after the simulated gastrointestinal digestion in vitro.

The upset plot of the common and differential peptides of the fermented milk samples prior to and after digestion is shown in Figure 2. In total, 1266 peptides were detected in the samples, with 846 peptides found in the samples before digestion and 641 peptides found in the samples after digestion. The peptides ranged from 232 to 384 in the fermented milk samples, with 88 detected in skim milk. Among these peptides, 20–51% of the peptides originated from β-casein, followed by αs1-casein, αs2-casein, and κ-casein in general, as reported previously [17].

Figure 2a shows variation among the strains in the capacity of the generation of differential peptides, with the most abundant differential peptides (137) recorded in the fermented milk of ssp. *lactis* D11M188 and only 41 in that of ssp. *bulgaricus* DXJLHTS2M2.

After digestion, the number of peptides dramatically decreased in the samples of DXJLHTS2M2, 2038, and D11M188 (Figure 2b). However, a 16% increase was observed in the digested sample of ATCC12315. Furthermore, the number of peptides derived from β-casein decreased with those from other proteins increased in the samples after digestion. The digested sample of ssp. *lactis* ATCC12315 possessed the most abundant differential peptides (87), while only 24 differential peptides were present in that of ssp. *bulgaricus* DXJLHTS2M2. In total, 28 peptides were shared by six digested samples, with 22 from the five digested samples of fermented milk.

### 3.3. Comparative Peptidomic Analysis of Fermented Milk at Subspecies Level

**Figure 3:**
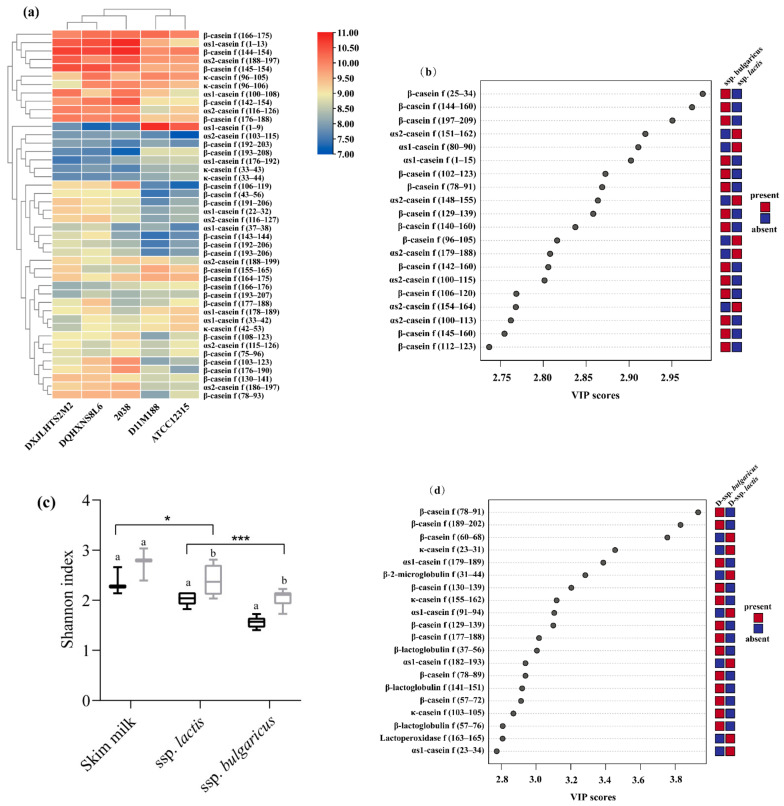
Comparative peptidomic analysis between the fermented milk of *Lactobacillus delbrueckii* ssp. *bulgaricus* and *L. delbrueckii* ssp. *lactis* before and after digestion. (**a**) Heat map of the common peptides in the samples. (**b**) Peptides with top 20 variable importance in projection (VIP) scores in fermented milk of *L. delbrueckii* ssp. *bulgaricus* and *L. delbrueckii* ssp. *lactis* according to partial least-squares discriminant analysis (PLS-DA). (**c**) Shannon index of the samples before (black) and after (grey) digestion. * and *** indicate significant difference (*p* < 0.05 and *p* < 0.001, respectively). Different lowercases letters indicate significant difference (*p* < 0.05) in the sample before and after digestion. (**d**) Peptides with top 20 VIP scores in digested samples of *L. delbrueckii* ssp. *bulgaricus* and *L. delbrueckii* ssp. *lactis* according to PLS-DA. D in front of samples name mean samples after digestion.

At *L. delbrueckii* species level, five fermented milks shared 44 peptides with a difference in abundance (Figure 3a). In total, 30 peptides were more abundant in the samples of ssp. *bulgaricus* such as β-casein f (106–119), β-casein f (103–123), and β-casein f (78–93), while 13 peptides were more abundant in the samples of ssp. *lactis* such as αs1-casein f (1–9), αs1-casein f (176–192), and β-casein f (193–208).

Comparison of the fermented samples was evaluated with PLS-DA, with R2X, R2Y, and Q2Y values of 0.979, 0.998, and 0.957, respectively (Figure S2), confirming the accuracy and predictability of the model. A total of 369 differential peptides were identified with VIP scores > 1, and the top 20 in the fermented samples are shown in Figure 3b. The peptides originating from β-casein were frequently present in the group of ssp. *bulgaricus*, whereas peptides derived from αs1-casein and αs2-casein were more likely to dominate in the group of ssp. *lactis* as previously reported [4,18].

Variation was observed for the common peptides at the subspecies level, with 61% of peptides derived from β-casein for the three samples of ssp. *bulgaricus* and 70% from αs1-, αs2-, and κ-casein for two samples of ssp. *lactis* (Tables S2 and S3). The significant difference of Shannon index between the samples from two subspecies indicated that the strains of *L. delbrueckii* ssp. *bulgaricus* displayed more intensive proteolysis of milk proteins during fermentation than those of *L. delbrueckii* ssp. *Lactis* and digestion appears to significantly reduce the richness of the peptide profile (*p* < 0.05) (Figure 3c).

Different LABs had different CEPs, which are responsible for the specificity for the substrate [19,20]. The CEP of *L. delbrueckii* ssp. *bulgaricus* was classified as PrtB [6], while PrtL of *L. delbrueckii* ssp. *lactis* has a unique amino acid substitution at position 222 for substrate specificity and different hydrolysis patterns of chromophoric peptides compared to PrtB [3]. A previous study indicated that both ssp. *bulgaricus* and ssp. *lactis* had similar hydrolysis activity against αs1-casein, αs2-casein, and β-casein based on the pattern of SDS-PAGE [3,18], which was inconsistent with the observation of the present study probably due to the variation in the experimental condition and analysis.

Comparison of the digested samples is shown in Figure 3d, in which R2X, R2Y, and Q2Y values (0.898, 0.991, and 0.688, respectively) are greater than 0.5 and could be used in recognition analysis (Figure S3). Similarly, a total of 322 differential peptides (VIP > 1) were identified. The peptides of the top 20 VIP scores indicated variation between subspecies (Figure 3d), particularly for β-casein f (78–91), β-casein f (189–202), and β-casein f (60–68), with the first two peptides presented in digested samples of ssp. *bulgaricus* and the latter in ssp. *lactis*.

### 3.4. Caseins Cleavage Pattern Based on Peptidomic Analysis

**Figure 4:**
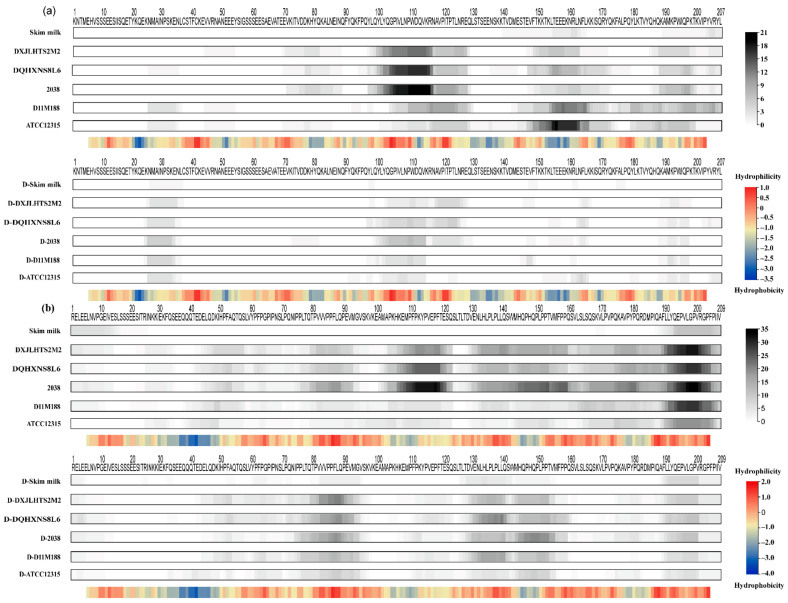
Heat map of αs2- and β-casein constructed from peptides identified in fermented and digested samples. Heat maps under the sequences indicated the frequency of the amino acids. A color is associated with each amino acid from white (no frequency) to black (high frequency). Heat maps under the plot indicated the hydrophilicity (red) and hydrophobicity (blue) of the corresponding sequence. (**a**) Heat maps constructed by peptides originated from αs2-casein in fermented and digested samples. (**b**) Heat maps constructed by peptides that originated from β-casein in fermented and digested samples. D in front of samples name means samples after digestion.

Variation on the cleavage pattern of αs2- and β-casein based on peptidomics was observed between the samples from the two subspecies in the heat map (Figure 4), in which ssp. *bulgaricus* displayed a stronger ability in hydrolyzing β-casein while ssp. *lactis* showed a preference to αs2-casein.

For αs2-casein, regions of (95–128) and (142–207) were extensively hydrolyzed and lead to the generation of a large number of peptides (Figure 4a). Specifically, ssp. *bulgaricus* produced notable peptides from the region (95–115), whereas ssp. *lactis* generated more peptides from the region (142–207). Among these peptides from αs2-casein, 13 were unique in the samples of ssp. *bulgaricus,* while 12 were unique in ssp. *lactis* (Tables S2 and S3). Besides the different regions preferred by two subspecies, there were common cleavage regions such as (114–128) and (185–199) shared in two subspecies, which generated seven peptides. However, the number of peptides released from αs2-casein was smaller than that from β-casein during milk fermentation, probably due to the fact that αs2-casein possessed more α-helix and was located in the interior of the casein micelle, which limits the accessibility of the CEPs and peptidases [21,22].

A previous study indicated that different CEPs may have varied preference to the cleavage regions. The cleavage sites of PrtH and PrtH2 on αs2-casein are located in the regions (1–25) and (97–162) [22]. H-strains with PrtS were able to hydrolyze αs2-casein, and most cleavage sites were confined to the region (159–207) [23]. Since the regions (3–18) and (126–207) of αs2-casein were hydrophilic, while region (78–109) was hydrophobic [23], it appeared that CEPs such as PrtP, PrtH, and PrtS preferred hydrophilic regions [20]. Our results indicate that ssp. *bulgaricus* (PrtB) might prefer the hydrophobic region while ssp. *lactis* (PrtL) might prefer the hydrophilic region.

Regarding β-casein, as it contains less secondary structure, it may be more accessible to hydrolysis and generate more peptides than the other caseins [22,24,25], which was confirmed by our results. The fermented samples of ssp. *bulgaricus* had more extensive hydrolysis in the regions (73–97), (100–120), and (130–209), whereas fermented samples of ssp. *lactis* had narrower hydrolysis in the region (160–209) with fewer peptides from β-casein (Figure 4b). Specifically, β-casein f (78–91), β-casein f (145–160), and β-casein f (195–206) are only presented in the samples of ssp. *bulgaricus,* which has been previously reported to produce bioactive peptides with antioxidant [26], anti-inflammatory [27], and ACE-inhibitory [28] activity in vitro, respectively.

It is worth noting that the C-terminal of the β-casein, a highly unstructured region, contributed a different pattern of hydrolysis between subspecies [22]. A previous study suggested that β-casein is an amphiphilic compound where the region (1–50) was hydrophilic and regions (153–175) and (187–209) were hydrophobic [23]. The results indicate that the CEPs of *L. delbrueckii* preferred hydrophobic regions of β-casein, similar to PrtS [20], with more extensive cleavage sites from ssp. *bulgaricus* than ssp. *lactis* during fermentation. No distinct profile between subspecies was observed for as1-casein (Figure S4) and κ-casein (Figure S5).

### 3.5. Pattern of Peptidomics of Fermented Milk after Digestion

As shown in Figure 4, the six digested samples had a very similar profile, which is consistent with our PCA analysis and the previous study [29]. As shown in Figure 4b, regions (95–115) and (142–207) of αs2-casein could be easily digested by pepsin and trypsin. Interestingly, after digestion, ssp. *bulgaricus* had some peptides remaining in the region (114–128) of αs2-casein, while ssp. *lactis* did not, which released a notable number of peptides through the fermentation of both subspecies and had a subspecies-based profile. In addition, the region (24–35) released several peptides in all fermented samples after digestion, such as αs2-casein f (25–33) and αs2-casein f (25–35). Alternatively, peptides from regions (73–97), (124–163), and (189–202) of β-casein still existed, while regions (100–120) and (164–188) diminished after the digestion of pepsin and trypsin (Figure 4b).

The long peptides, including the regions (202–209) of β-casein, were hydrolyzed into smaller peptides around arginine residues (position 202) at the C-terminal side (Figure 4b). This was probably due to the hydrolysis of trypsin as trypsin has been reported to generate peptides on the C-terminal side of lysine and arginine amino acid residues preferentially [30]. Otherwise, pepsin had an extensive cleavage specificity, which could cleave peptides with an aromatic acid on either side of the peptide bond, especially if the other residue is also an aromatic or a dicarboxylic amino acid, except for valine, alanine, or glycine linkages [31], but no distinct pattern could be found by the hydrolysis activity of pepsin.

### 3.6. Bioactive Peptides in Fermented Milk after Digestion

As shown in Table 1, peptides 173 to 271 were detected in the digested samples of the skim milk and fermented milk samples. The results suggested that the fermentation of *L. delbrueckii* generally led to the generation of more digestion-resistant and bioactive peptides in the fermented milk samples than in the skim milk [29]. The fermented milk of DQHXNS8L6 had the most abundant digestion-resistant peptides, defined as being present in both undigested and digested samples. Specifically, a total of 11 common digestion-resistant peptides were detected in all the fermented milk samples, among which 4 peptides were reported with the bioactivity of being antioxidant, ACE-inhibitory and immunomodulatory (Table 2), and 6 of them were recorded both in the fermented and digested samples including αs2-casein f (189–197), β-casein f (130–141), f (142–154), (145–154), and κ-casein f (96–105), f (96–106).

The bioactivity of the peptides is shown in Table 3, with 12 bioactive peptides from whey proteins. In general, 16 to 37 bioactive peptides were identified in the six digested samples, and DQHXNS8L6 and D11M188 had the most abundant bioactive peptides. Thirteen bioactive peptides originated from αs1-casein, with three, four, and six bioactive peptides from the regions (23–40), (90–94), and (146–199), respectively. Among them, antioxidant αs1-casein f (39–40) and αs1-casein f (146–149) were unique in the digested samples of DXJLHTS2M2 and DQHXNS8L6, whereas antimicrobial αs1-casein f (180–193) was shared in all the digested samples. For αs2-casein, only one bioactive peptide, αs2-casein f (25–32), was detected in the digested samples of 2038, D11M188, and ATCC12315. However, the most abundant bioactive peptides were generated from the regions (52–86), (106–160), and (191–209) of β-casein, with ten, nine, and seven bioactive peptides present. Specifically, β-casein f (84–86), also known as VPP for multiple bioactivities, was detected in the digested samples of 2038 and D11M188.

## 4. Conclusions

In the present study, a difference was observed in the peptidomic feature of fermented milks before and after the simulated gastrointestinal digestion in vitro between subspecies, mainly originating from the hydrolysis of αs2- and β-casein. Regarding αs2-casein, ssp. *bulgaricus* generated notable peptides in the region (95–115), whereas ssp. *lactis* formed more peptides and a more distributed pattern from the region (142–207). For β-casein, ssp. *bulgaricus* had more extensive cleavage distribution, including regions (73–97), (100–120), and (130–209), whereas ssp. *lactis* had a narrower distribution in the region (160–209) with fewer peptides. After digestion, *L. delbrueckii* ssp. *bulgaricus* DQHXNS8L6 had the most peptides, peptides resisting digestion, bioactive peptides, and short-sized peptides, together with robust growth and acidification capacity.

## Figures and Tables

**Table 1:** General peptidomic characteristics of skim milk and fermented milk samples of *Lactobacillus delbrueckii* ssp. *bulgaricus* and *L. delbrueckii* ssp. *lactis* after the simulated gastrointestinal digestion in vitro.

Parameter	Skim Milk	DXJLHTS2M2	DQHXNS8L6	2038	D11M188	ATCC12315
Total peptides	185	173	293	219	219	271
Specific peptides	83	24	77	34	29	87
Anti-digestion peptides	7	57	87	76	51	42
Bioactive peptides	16	22	37	26	35	28
Short-sized peptides ^1^	90	49	155	61	85	155
Medium-sized peptides ^2^	69	106	114	125	102	87
Long-sized peptides ^3^	25	17	21	32	29	27

^1^ Short-sized peptides were defined as peptides with 2–5 amino acids, ^2^ medium-sized peptides were defined as peptides with 6–15 amino acids, ^3^ long-sized peptides were defined as peptides with 16–25 amino acids.

**Table 2:** Common digestion-resistant peptides identified in fermented milk of *Lactobacillus delbrueckii* ssp. *bulgaricus* and *L. delbrueckii* ssp. *lactis*.

Fragment	Bioactivity	Fermented Milks	D-Fermented Milks	Skim Milk	D-Skim Milk
αs1-casein f (21–22) ^1^		√	√		√
αs1-casein f (178–189)		√	√		√
**αs2-casein f (189–197)** ^2^		√	√		
β-casein f (78–93)		√	√		√
**β-casein f (130–141)**		√	√		
**β-casein f (142–154)**	Antioxidant	√	√		
β-casein f (144–154)		√	√		√
**β-casein f (145–154)**	ACE-inhibitory	√	√		
β-casein f (192–209)	Immunomodulatory	√	√	√	√
**κ-casein f (96–105)**		√	√		
**κ-casein f (96–106)**	Antioxidant	√	√		

^1^ The positions of fragments listed refer to search result of Maxquant, and the underline means that the fragments of peptides were not the unique in milk proteins. ^2^ Fragments listed with bold fonts mean that the peptides were generated during fermentation and could resist the digestion process. D in front of samples name means samples after digestion.

**Table 3:** Bioactive peptides identified in the digested skim milk and fermented milk samples of *Lactobacillus delbrueckii* ssp. *bulgaricus* and *L. delbrueckii* ssp. *lactis*.

Fragment	Bioactivity ^2^	Skim Milk	DXJLHTS2M2	DQHXNS8L6	2038	D11M188	ATCC12315
β-lactoglobulin f (31–32) ^1^	1			√	√	√	
β-lactoglobulin f (41–60)	10		√		√		
β-lactoglobulin f (102–104)	1, 6	√	√	√	√	√	√
β-lactoglobulin f (125–135)	5, 8		√	√	√		
β-lactoglobulin f (104–105)	1, 4	√					√
Lactotransferrin f (165–166)	1					√	
Lactotransferrin f (166–167)	1, 3, 4, 8	√					√
Lactotransferrin f (318–319)	1			√			
serum albumin f (112–113)	8	√		√			
serum albumin f (221–222)	1	√	√	√	√	√	√
α-lactalbumin f (50–51)	1			√		√	
α-lactalbumin f (104–105)	1, 8	√	√	√	√	√	√
αs1-casein f (23–34)	1				√	√	√
αs1-casein f (28–34)	1						√
αs1-casein f (39–40)	6		√				
αs1-casein f (90–92)	1, 6						√
αs1-casein f (91–92)	1	√	√	√			√
αs1-casein f (91–93)	6, 12			√			
αs1-casein f (91–94)	1, 6					√	√
αs1-casein f (146–149)	6			√			
αs1-casein f (165–166)	1						√
αs1-casein f (176–192)	6					√	√
αs1-casein f (180–193)	5	√	√	√	√	√	√
αs1-casein f (194–199)	1, 5			√			
αs1-casein f (198–199)	1			√			
αs2-casein f (25–32)	1				√	√	√
β-casein f (52–53)	1	√					
β-casein f (59–68)	1, 6	√				√	
β-casein f (60–63)	2, 14					√	
β-casein f (60–68)	1, 6, 8					√	√
β-casein f (61–63)	1					√	
β-casein f (61–68)	1						√
β-casein f (70–72)	8			√			
β-casein f (73–89)	1			√	√		
β-casein f (78–91)	6		√	√	√		
β-casein f (84–86)	1, 4, 6, 9, 13, 15, 16				√	√	
β-casein f (106–113)	5		√		√	√	
β-casein f (130–140)	1		√	√	√	√	
β-casein f (132–140)	1		√	√		√	
β-casein f (135–137)	8		√	√		√	√
β-casein f (142–154)	6		√	√	√	√	√
β-casein f (143–154)	1, 4		√	√	√	√	√
β-casein f (145–154)	1		√	√	√	√	√
β-casein f (145–160)	1, 4			√	√	√	
β-casein f (151–153)	1					√	
β-casein f (169–176)	6				√		
β-casein f (191–193)	4, 6, 11			√		√	√
β-casein f (191–202)	1	√	√	√	√	√	√
β-casein f (191–209)	1	√					
β-casein f (192–202)	1, 4	√	√	√	√	√	√
β-casein f (192–209)	11	√	√	√	√	√	√
β-casein f (193–202)	1, 4, 6, 7, 11	√	√	√	√	√	
β-casein f (193–209)	1, 5, 7, 11	√	√	√	√	√	√
κ-casein f (7–8)	1			√			
κ-casein f (14–17)	1			√		√	√
κ-casein f (30–32)	5, 6			√		√	√
κ-casein f (58–60)	8			√			
κ-casein f (96–106)	6		√	√	√	√	√
κ-casein f (97–106)	6			√	√	√	√
κ-casein f (150–151) ^3^	1			√			

^1^ The positions of fragments listed referred to MDPDB, and the underline means that the fragments of peptides were not unique in milk protein. ^2^ Bioactivity represented by numbers is as follows: 1, ACE-inhibitory; 2, Anticancer; 3, Antihypertensive; 4, Anti-inflammatory; 5, Antimicrobial; 6, Antioxidant; 7, Antithrombotic; 8, DPP-IV inhibitory; 9, Enhance insulin signaling; 10, Hypocholesterolemic; 11, Immunomodulatory; 12, Improves cognitive decline; 13, Nitric oxide liberation; 14, Opioid; 15, Reduces bone loss; 16, Stimulates trabecular bone growth. ^3^ The derived protein was κ-casein Genetic Variant F1.

## Data Availability

The datasets generated for this study are available on request to the corresponding author.

## Supplementary Materials

The following are available online at https://www.mdpi.com/article/10.3390/foods10123028/s1, Figure S1: Peptide content of skim milk and fermented milks by *Lactobacillus delbrueckii* ssp. *bulgaricus* and *Lactobacillus delbrueckii* ssp. *lactis* after 12 h of fermentation at 37 °C before (black) and after (gray) simulated gastrointestinal digestion in vitro; Figure S2: PLS-DA score plot for fermented milks peptidomic of *Lactobacillus delbrueckii* ssp. *bulgaricus* and *Lactobacillus delbrueckii* ssp. *Lactis*; Figure S3: PLS-DA score plot for digested samples peptidomic of *Lactobacillus delbrueckii* ssp. *bulgaricus* and *Lactobacillus delbrueckii* ssp. *Lactis*; Figure S4: αs1-casein heat maps constructed by peptides present in all undigested and digested samples, respectively. Heat maps under the sequences indicated the frequency of the amino acids; Figure S5: κ-casein heat maps constructed by peptides present in all undigested and digested samples, respectively; Table S1: pH value and viable count of fermented milks by *Lactobacillus delbrueckii* ssp. *bulgaricus* and *Lactobacillus delbrueckii* ssp. *lactis* after 12 h fermentation under 37 °C. Table S2: Common peptides presented in fermented milk samples of *Lactobacillus delbrueckii* ssp. *Bulgaricus*; Table S3: Common peptides presented in fermented milk samples of *Lactobacillus delbrueckii* ssp. *Lactis*.

## References

[1] Weiss N., Schillinger U., Kandler O. (1983). Lactobacillus lactis, Lactobacillus leichmannii and Lactobacillus bulgaricus, Subjective Synonyms of Lactobacillus delbrueckii, and Description of Lactobacillus delbrueckii subsp. lactis comb. nov. and Lactobacillus delbrueckii subsp. bulgaricus comb. nov. Syst. Appl. Microbiol..

[2] El Kafsi H., Binesse J., Loux V., Buratti J., Boudebbouze S., Dervyn R., Kennedy S., Galleron N., Quinquis B., Batto J.-M. (2014). Lactobacillus delbrueckii ssp. lactis and ssp. bulgaricus: A chronicle of evolution in action. BMC Genom..

[3] Villegas J.M., Brown L., Savoy de Giori G., Hebert E.M. (2015). Characterization of the mature cell surface proteinase of Lactobacillus delbrueckii subsp. lactis CRL 581. Appl. Microbiol. Biotechnol..

[4] Laloi P., Atlan D., Blanc B., Gilbert C., Portalier R. (1991). Cell-wall-associated proteinase of Lactobacillus–delbrueckii subsp. bulgaricus CNRZ 397: Differential extraction, purification and properties of the enzyme. Appl. Microbiol. Biotechnol..

[5] Tsakalidou E., Anastasiou R., Vandenberghe I., Van Beeumen J., Kalantzopoulos G. (1999). Cell-Wall-Bound Proteinase of Lactobacillus delbrueckii subsp. lactis ACA-DC 178: Characterization and Specificity for β-Casein. Appl. Environ. Microbiol..

[6] Gilbert C., Atlan D., Blanc B., Portalier R., Germond J.E., Lapierre L., Mollet B. (1996). A new cell surface proteinase: Sequencing and analysis of the prtB gene from Lactobacillus delbrueckii subsp bulgaricus. J. Bacteriol..

[7] Nongonierma A.B., Fitzgerald R.J. (2015). The scientific evidence for the role of milk protein-derived bioactive peptides in humans: A Review. J. Funct. Foods.

[8] Park Y.W., Nam M.S. (2015). Bioactive Peptides in Milk and Dairy Products: A Review. Food Sci. Anim. Resour..

[9] Nielsen S.D.-H., Beverly R.L., Qu Y., Dallas D.C. (2017). Milk bioactive peptide database: A comprehensive database of milk protein-derived bioactive peptides and novel visualization. Food Chem..

[10] Nakamura Y., Yamamoto N., Sakai K., Okubo A., Yamazaki S., Takano T. (1995). Purification and Characterization of Angiotensin I-Converting Enzyme Inhibitors from Sour Milk. J. Dairy Sci..

[11] Nakamura Y., Yamamoto N., Sakai K., Takano T. (1995). Antihypertensive Effect of Sour Milk and Peptides Isolated from It That are Inhibitors to Angiotensin I-Converting Enzyme. J. Dairy Sci..

[12] Hata Y., Yamamoto M., Ohni M., Nakajima K., Nakamura Y., Takano T. (1996). A placebo-controlled study of the effect of sour milk on blood pressure in hypertensive subjects. Am. J. Clin. Nutr..

[13] Ohsawa K., Satsu H., Ohki K., Enjoh M., Takano T., Shimizu M. (2008). Producibility and Digestibility of Antihypertensive β-Casein Tripeptides, Val-Pro-Pro and Ile-Pro-Pro, in the Gastrointestinal Tract: Analyses Using anin VitroModel of Mammalian Gastrointestinal Digestion. J. Agric. Food Chem..

[14] Wang J., Jiang Y., Yu P., Lee Y.K., Liu X., Zhao J., Zhang H., Chen W. (2021). Effect of carbon catabolite repression on lactose and galactose catabolism in Lacticaseibacillus paracasei. Food Biosci..

[15] Wu N., Xu W., Liu K., Xia Y. (2019). Shuangquan Angiotensin-converting enzyme inhibitory peptides from Lactobacillus delbrueckii QS306 fermented milk. J. Dairy Sci..

[16] Quirós A., Dávalos A., Lasunción M.A., Ramos M., Recio I. (2008). Bioavailability of the antihypertensive peptide LHLPLP: Transepithelial flux of HLPLP. Int. Dairy J..

[17] Hao X., Yang W., Zhu Q., Zhang G., Zhang X., Liu L., Li X., Hussain M., Ni C., Jiang X. (2021). Proteolysis and ACE-inhibitory peptide profile of Cheddar cheese: Effect of digestion treatment and different probiotics. LWT.

[18] Kliche T., Li B., Bockelmann W., Habermann D., Klempt M., De Vrese M., Wutkowski A., Clawin-Raedecker I., Heller K.J. (2017). Screening for proteolytically active lactic acid bacteria and bioactivity of peptide hydrolysates obtained with selected strains. Appl. Microbiol. Biotechnol..

[19] Liu M., Bayjanov J.R., Renckens B., Nauta A., Siezen R.J. (2010). The proteolytic system of lactic acid bacteria revisited: A genomic comparison. BMC Genom..

[20] Ji D., Ma J., Xu M., Agyei D. (2020). Cell-envelope proteinases from lactic acid bacteria: Biochemical features and biotechnological applications. Compr. Rev. Food Sci. Food Saf..

[21] Farrell H.M., Malin E.L., Brown E.M., Mora-Gutierrez A. (2009). Review of the chemistry of alphaS2-casein and the generation of a homologous molecular model to explain its properties. J. Dairy Sci..

[22] Sadat-Mekmene L., Jardin J., Corre C., Mollé D., Richoux R., Delage M.-M., Lortal S., Gagnaire V. (2011). Simultaneous Presence of PrtH and PrtH2 Proteinases in Lactobacillus helveticus Strains Improves Breakdown of the Pure α s1 -Casein. Appl. Environ. Microbiol..

[23] Miclo L., Roux E., Genay M., Brusseaux E., Poirson C., Jameh N., Perrin C., Dary A. (2012). Variability of hydrolysis of beta-, alphas1-, and alphas2-caseins by 10 strains of Streptococcus thermophilus and resulting bioactive peptides. J. Agric. Food Chem..

[24] Holt C., Sawyer L. (1993). Caseins as rheomorphic proteins: Interpretation of primary and secondary structures of the αS1-, β- and κ-caseins. J. Chem. Soc. Faraday Trans..

[25] Kumosinski T.F., Brown E.M., Farrell H.M. (1993). Three-dimensional molecular modeling of bovine caseins: An energy-minimized beta-casein structure. J. Dairy Sci..

[26] Sabeena F.K.H., Baron C.P., Nielsen N.S., Otte J., Jacobsen C. (2010). Antioxidant activity of yoghurt peptides: Part 2–Characterisation of peptide fractions. Food Chem..

[27] Adams C., Sawh F., Green-Johnson J.M., Jones T.H., Strap J.L. (2020). Characterization of casein-derived peptide bioactivity: Differential effects on angiotensin-converting enzyme inhibition and cytokine and nitric oxide production. J. Dairy Sci..

[28] Hayes M., Stanton C., Slattery H., O’Sullivan O., Hill C., Fitzgerald G.F., Ross R.P. (2007). Casein Fermentate of Lactobacillus animalis DPC6134 Contains a Range of Novel Propeptide Angiotensin-Converting Enzyme Inhibitors. Appl. Environ. Microbiol..

[29] Sanchez-Rivera L., Diezhandino I., Gomez-Ruiz J.A., Fresno J.M., Miralles B., Recio I. (2014). Peptidomic study of Spanish blue cheese (Valdeon) and changes after simulated gastrointestinal digestion. Electrophoresis.

[30] Baird T.T., Craik C.S., Brenner S., Miller J.H. (2001). Trypsin. Encyclopedia of Genetics.

[31] Sweeney P.J., Walker J.M., Burrell M.M. (1993). Proteolytic Enzymes for Peptide Production. Enzymes of Molecular Biology.

