# Supplementary material for: Comparative Peptidomics Analysis of Fermented Milk by Lactobacillus delbrueckii ssp. bulgaricus and Lactobacillus delbrueckii ssp. lactis
Source: Foods. 2021 Dec 6;10(12):3028. doi: 10.3390/foods10123028 (PMC8701751; doi:10.3390/foods10123028)
Supplement: Supplementary file 1 [file foods-10-03028-s001.zip › foods-1468903-supplementary.pdf]

Supplementary Materials

Table S1. The pH value and viable count of fermented milk samples by *Lactobacillus delbrueckii* ssp. *bulgaricus* and *L. delbrueckii* ssp. *lactis* after 12 h fermentation under 37 ℃.

samples	DXJLHTS2M2	DQHXNS8L6	2038	D11M188	ATCC12315
pH	4.40±0.02 ^{b}	4.39±0.01 ^{b}	3.90±0.04 ^{a}	4.30±0.02 ^{b}	5.76±0.02 ^{c}
ΔViable count Lg (CFU/mL)	2.22±0.08 ^{b}	2.75±0.02 ^{a}	2.71±0.05 ^{a}	2.29±0.02 ^{b}	1.84±0.01 ^{c}

Data marked with different lowercase superscripts in the same row indicated significant differences between samples ($p < 0.05$).

Table S2. Common peptides presented in fermented milk samples of *Lactobacillus delbrueckii* ssp. *bulgaricus*.

Fragment	Sequence
BTN1A1 f (179–190) [1]	THRGEEFPSMSE
GLYCAM1 f (19–43) [2]	ILNKPEDETHLEAQPTDASAQFIRN
GLYCAM1 f (51–60)	LSKEPSISRE
GLYCAM1 f (61–71)	DLISKEQIVIR
GLYCAM1 f (63–71)	ISKEQIVIR
LYST f (980–982) [3]	YRL
αs1-casein f (1–8)	RPKHPIKH
αs1-casein f (2–3)	PK
αs1-casein f (10–20)	GLPQEVLNENL
αs1-casein f (10–21)	GLPQEVLNENLL
αs1-casein f (25–32)	VAPFPEVF
αs1-casein f (35–42)	EKVNELSK
αs1-casein f (80–89)	HIQKEDVPSE
αs1-casein f (81–90)	IQKEDVPSER
αs1-casein f (98–105)	LLRLKKYK
αs1-casein f (109–119)	LEIVPNSAEER
αs1-casein f (109–121)	LEIVPNSAEERLH
αs1-casein f (100–105)	RLKKYK
αs1-casein f (110–119)	EIVPNSAEER
αs1-casein f (115–123)	SAEERLHSM
αs1-casein f (173–189)	YTDAPSFSDIPNPIGSE
αs1-casein f (174–189)	TDAPSFSDIPNPIGSE
αs1-casein f (176–191)	APSFSDIPNPIGSENS
αs1-casein f (177–189)	PSFSDIPNPIGSE
αs2-casein f (25–33)	NMAINPSKE
αs2-casein f (108–122)	PWDQVKRNAVPITPT
αs2-casein f (143–150)	STEVFTKK
αs2-casein f (143–152)	STEVFTKKTK
αs2-casein f (148–155)	TKKTKLTE
αs2-casein f (153–164)	LTEEEKNRLNFL
αs2-casein f (156–165)	EEKNRLNFLK
αs2-casein f (157–165)	EKNRLNFLK
αs2-casein f (179–187)	YLKTVYQHQ
αs2-casein f (179–188)	YLKTVYQHQK
αs2-casein f (179–189)	YLKTVYQHQKA
αs2-casein f (191–199)	KPWIQPKTK
β-casein f (37–48)	EQQQTEDELQDK
β-casein f (38–48)	QQQTEDELQDK
β-casein f (40–48)	QTEDELQDK
β-casein f (96–105)	SKVKEAMAPK
β-casein f (133–134)	LH
β-casein f (176–187)	KAVPYPQRDMPI
β-casein f (191–208)	LLYQEPVLGPVRGPFPII
β-casein f (192–207)	LYQEPVLGPVRGPFPI
β-casein f (192–208)	LYQEPVLGPVRGPFPII
κ-casein f (149–160)	SPEVIESPPEIN
κ-casein f (150–160)	PEVIESPPEIN
κ-casein f (32–43)	LSRYPSYGLNYY
κ-casein f (34–43)	RYPSYGLNYY
κ-casein f (34–44)	RYPSYGLNYYQ

Fragments underlined mean that the fragments of the peptides were not unique in milk proteins. [1] LYST, Lysosomal-trafficking regulator; [2] PYGL, Glycogen phosphorylase, liver form.

Table S3. Common peptides presented in fermented milk samples of *Lactobacillus delbrueckii* ssp. *lactis*.

Fragment	Sequence
BTN1A1 f (179–190) [1]	THRGEEFPSMSE
GLYCAM1 f (19–43) [2]	ILNKPEDETHLEAQPTDASAQFIRN
GLYCAM1 f (51–60)	LSKEPSISRE
GLYCAM1 f (61–71)	DLISKEQIVIR
GLYCAM1 f (63–71)	ISKEQIVIR
LYST f (980–982) [3]	YRL
αs1-casein f (1–8)	RPKHPIKH
αs1-casein f (2–3)	PK
αs1-casein f (10–20)	GLPQEVLNENL
αs1-casein f (10–21)	GLPQEVLNENLL
αs1-casein f (25–32)	VAPFPEVF
αs1-casein f (35–42)	EKVNELSK
αs1-casein f (80–89)	HIQKEDVPSE
αs1-casein f (81–90)	IQKEDVPSER
αs1-casein f (98–105)	LLRLKKYK
αs1-casein f (109–119)	LEIVPNSAEER
αs1-casein f (109–121)	LEIVPNSAEERLH
αs1-casein f (100–105)	RLKKYK
αs1-casein f (110–119)	EIVPNSAEER
αs1-casein f (115–123)	SAEERLHSM
αs1-casein f (173–189)	YTDAPSFSDIPNPIGSE
αs1-casein f (174–189)	TDAPSFSDIPNPIGSE
αs1-casein f (176–191)	APSFSDIPNPIGSENS
αs1-casein f (177–189)	PSFSDIPNPIGSE
αs2-casein f (25–33)	NMAINPSKE
αs2-casein f (108–122)	PWDQVKRNAVPITPT
αs2-casein f (143–150)	STEVFTKK
αs2-casein f (143–152)	STEVFTKKTK
αs2-casein f (148–155)	TKKTKLTE
αs2-casein f (153–164)	LTEEEKNRLNFL
αs2-casein f (156–165)	EEKNRLNFLK
αs2-casein f (157–165)	EKNRLNFLK
αs2-casein f (179–187)	YLKTVYQHQ
αs2-casein f (179–188)	YLKTVYQHQK
αs2-casein f (179–189)	YLKTVYQHQKA
αs2-casein f (191–199)	KPWIQPKTK
β-casein f (37–48)	EQQQTEDELQDK
β-casein f (38–48)	QQQTEDELQDK
β-casein f (40–48)	QTEDELQDK
β-casein f (96–105)	SKVKEAMAPK
β-casein f (133–134)	LH
β-casein f (176–187)	KAVPYPQRDMPI
β-casein f (191–208)	LLYQEPVLGPVRGPFPII
β-casein f (192–207)	LYQEPVLGPVRGPFPI
β-casein f (192–208)	LYQEPVLGPVRGPFPII
κ-casein f (149–160)	SPEVIESPPEIN
κ-casein f (150–160)	PEVIESPPEIN
κ-casein f (32–43)	LSRYPSYGLNYY
κ-casein f (34–43)	RYPSYGLNYY
κ-casein f (34–44)	RYPSYGLNYYQ

Fragments underlined mean that the fragments of the peptides were not unique in milk proteins. [1] BTN1A1, Butyrophilin subfamily 1 member A1; [2] GLYCAM1, Glycosylation-dependent cell adhesion molecule 1; [3] LYST, Lysosomal-trafficking regulator.

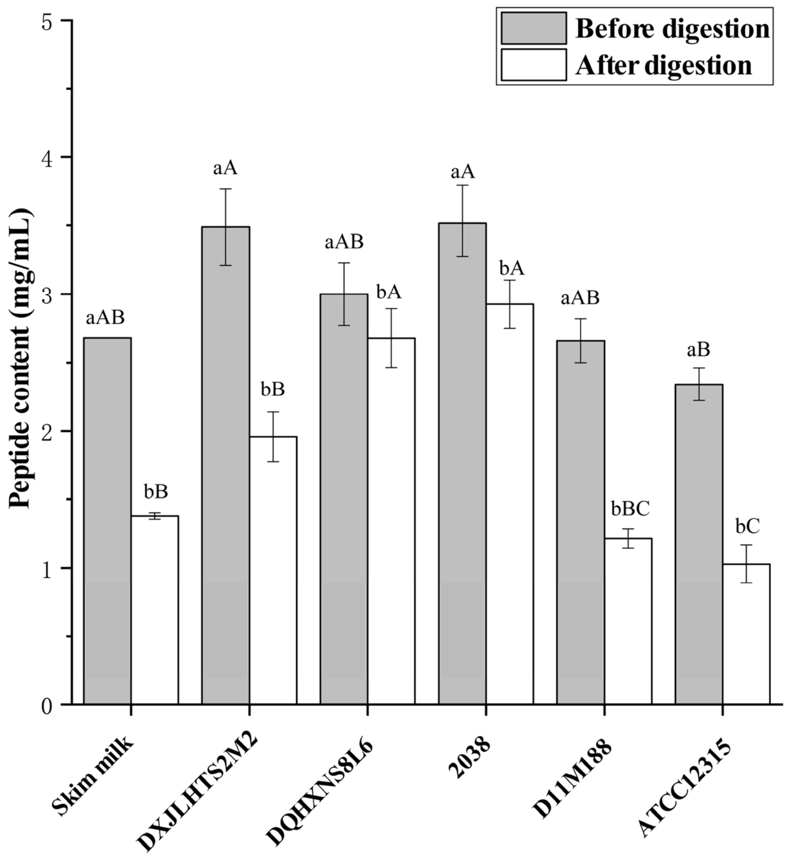

Figure S1. Peptide content of skim milk and fermented milk samples by *Lactobacillus delbrueckii* ssp. *bulgaricus* and *L. delbrueckii* ssp. *lactis* after 12h of fermentation at 37℃ before (black) and after (gray) simulated gastrointestinal digestion in vitro. Data marked with different lowercase superscripts indicated significant differences in the same sample before and after digestion ($P < 0.05$), and different uppercase superscripts indicated significant differences in different samples prior to digestion or after digestion ($P < 0.05$).

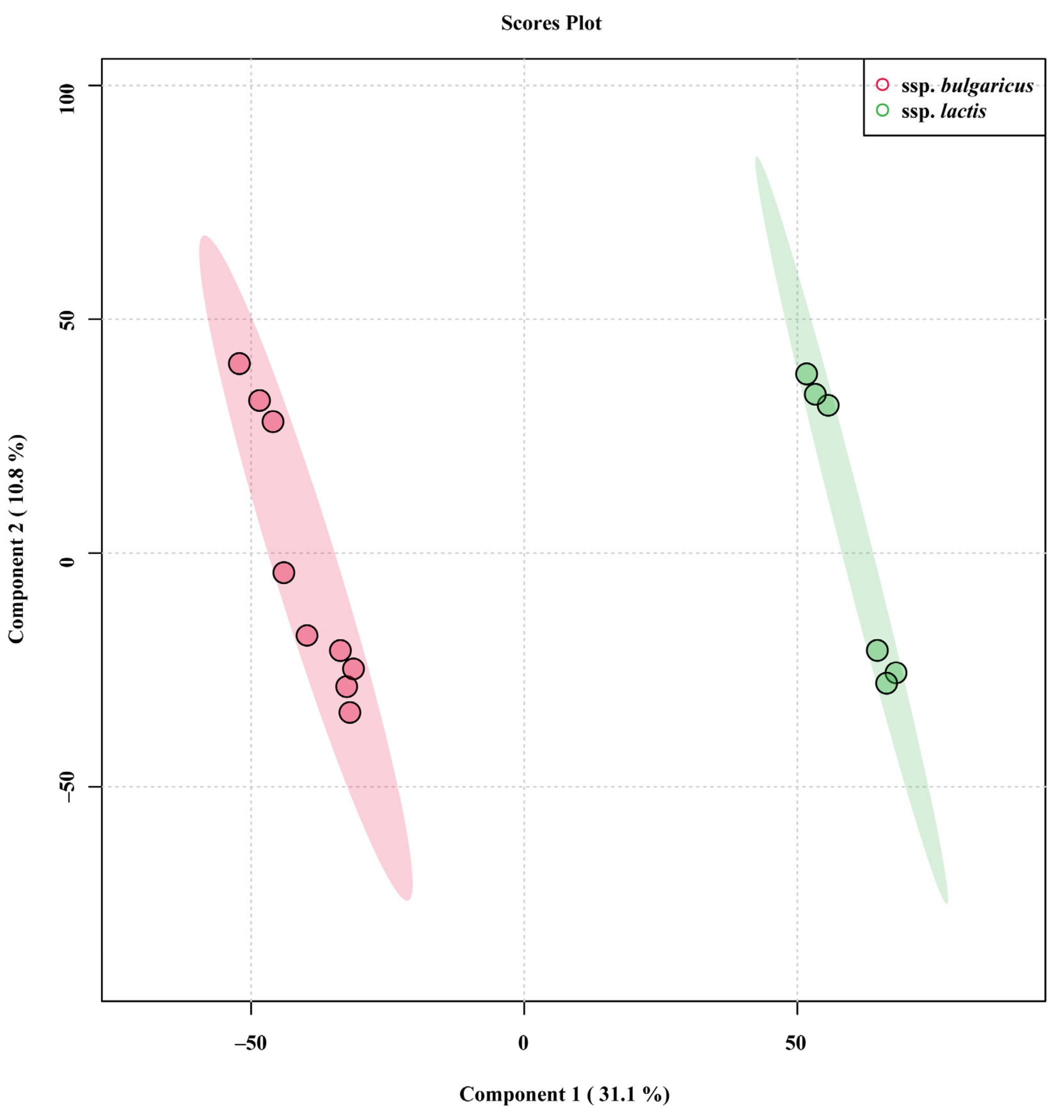

Figure S2. PLS-DA score plot based on peptidomics of fermented milk samples of *Lactobacillus delbrueckii* ssp. *bulgaricus* and *L. delbrueckii* ssp. *lactis.*

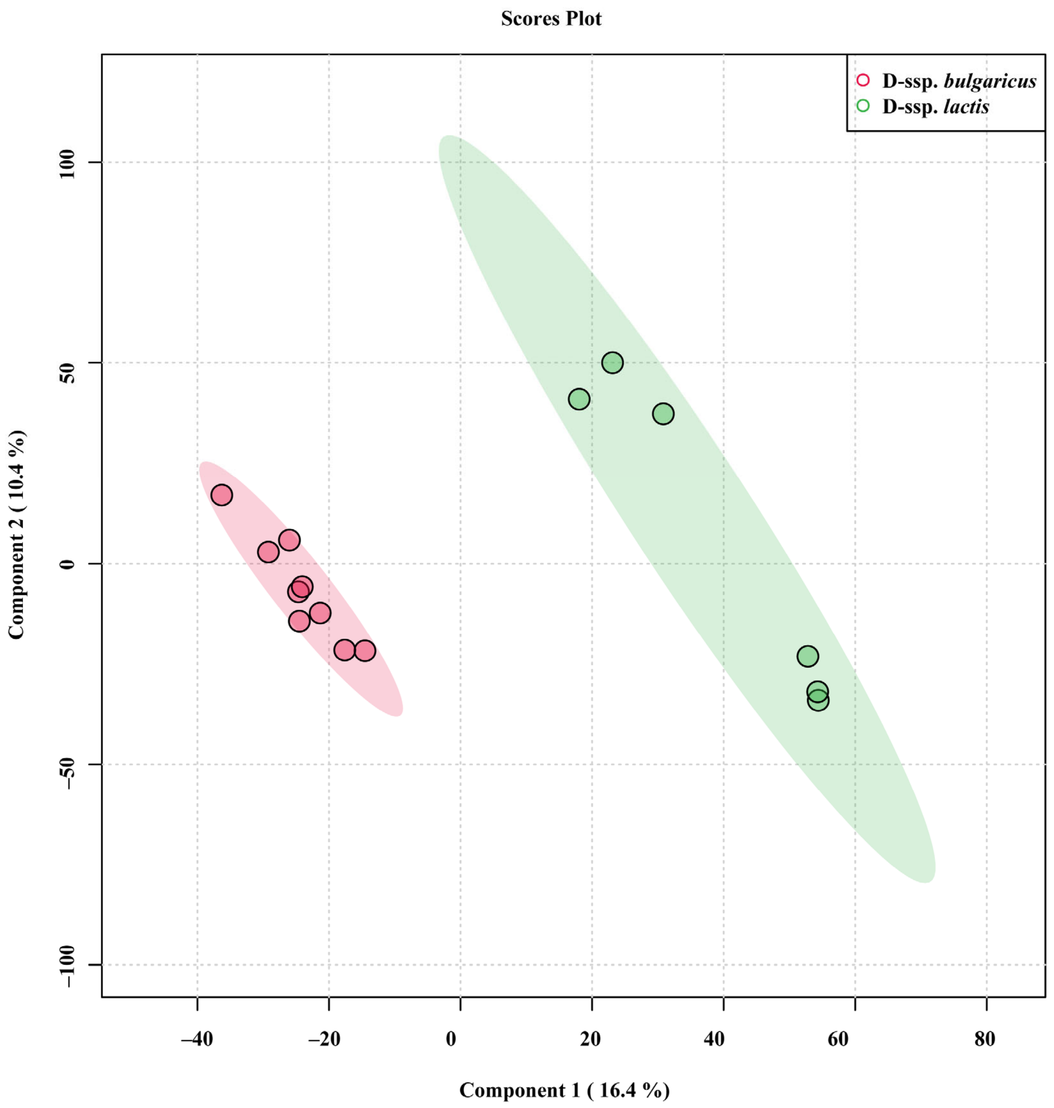

Figure S3. PLS-DA score plot based on peptidomics of the digested fermented milk samples of *Lactobacillus delbrueckii* ssp. *bulgaricus* and *L. delbrueckii* ssp. *lactis*.

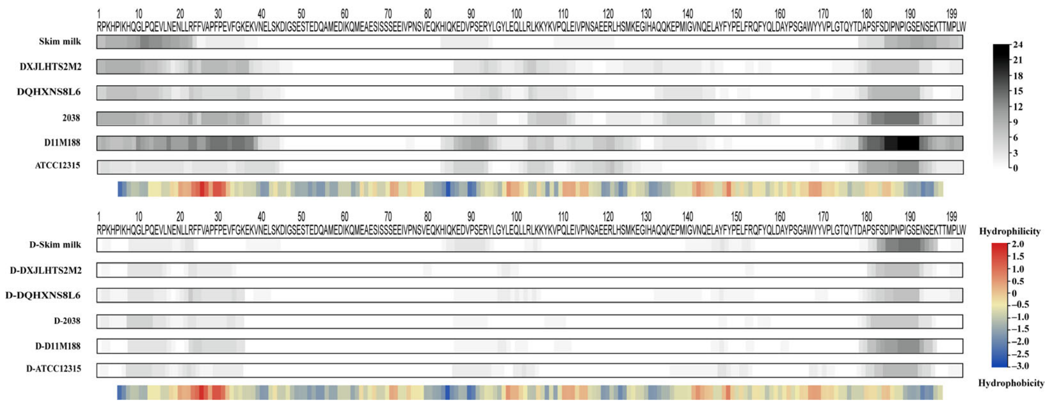

Figure S4. Heat map of αs1-casein constructed from peptides identified in fermented and digested samples. Heat maps under the sequences indicated the frequency of the amino acids. A color was associated to each amino acid from white (no frequency) to black (high frequency). Heat maps under the plot indicated the hydrophilicity (red) and hydrophobicity (blue) of the corresponding sequence.

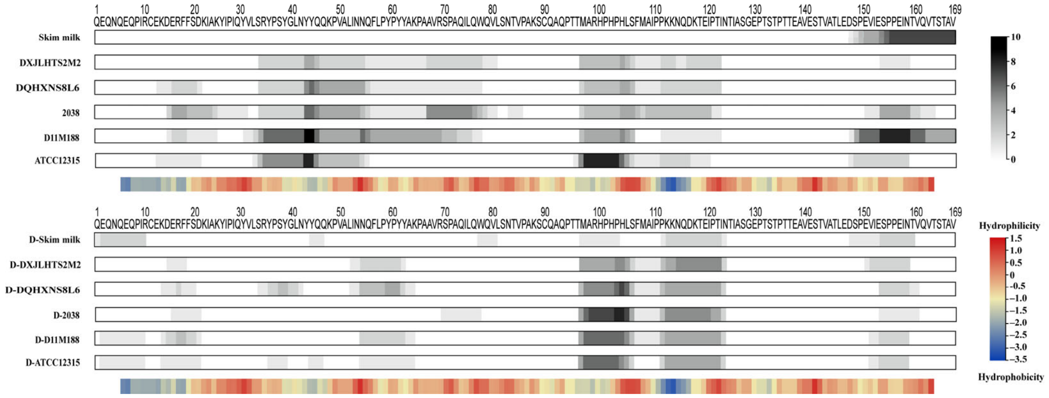

Figure S5. Heat map of κ-casein constructed from peptides identified in fermented and digested samples. Heat maps under the sequences indicated the frequency of the amino acids. A color was associated to each amino acid from white (no frequency) to black (high frequency). Heat maps under the plot indicated the hydrophilicity (red) and hydrophobicity (blue) of the corresponding sequence.
